# Anemia-Related Red Flags as Distinctive Early Indicators for Colorectal Cancer Diagnosis in Older Adults

**DOI:** 10.3390/jcm15114039

**Published:** 2026-05-23

**Authors:** Aviad Bachar, Zohar Levi, Doron Boltin, Hadar Edelman-Klapper, Ram Dickman, Tsachi Tsadok Perets, Rachel Gingold-Belfer

**Affiliations:** 1 Gastroenterology Division, Rabin Medical Center, Beilinson Hospital, Petach Tikva 4941492, Israel; 2 Gray School of Medicine, Gray Faculty of Medical and Health Sciences, Tel Aviv University, Tel Aviv 6997801, Israel; 3 Department of Digital Medical Technologies, Holon Institute of Technology, Holon 5810201, Israel

**Keywords:** anemia, colorectal cancer, diagnosis, older adults, iron deficiency

## Abstract

**Background:** Anemia is an important clinical sign which often points to a serious and possibly treatable medical condition. We aimed to investigate the frequency of anemia long before colorectal cancer (CRC) surgeries among older adults aged ≥ 75 years (group 2), compared to patients aged 50–75 years (group 1). Participants and setting: Consecutive patients operated for CRC in our center who belonged to the health maintenance organization (HMO) at least 36 months before their diagnosis were included. **Methods:** Laboratory data were extracted from the HMO database. Anemia and severe anemia were defined as Hb < 14 g/dL and <12 g/dL for males and <12 g/dL and <10 g/dL for females, respectively. Low serum iron was defined as below 45 mcg/dL. **Results:** 647 patients were identified, including 277 (42.8%) ≥75 years (mean age 80.7 ± 4.1 y) and 370 (57.1%), aged 50–75 years (mean 65.8 ± 6.5 y). CRC was more commonly diagnosed following a presentation of iron deficiency anemia in group 2 (20.6% vs. 11.6%, *p* = 0.002), while positive fecal occult blood test was more frequent among the younger group. Anemia and severe anemia ≥ 1 year before CRC diagnosis were observed in 156 (56.3%) and 56 (20.2%) patients of group 2 compared to 142 (38.4%) and 38 (10.3%) of patients from group 1 (*p* < 0.001 and *p* = 0.001, respectively). A low iron level ≥ 1 year before diagnosis was observed in 49 (17.6%) patients in group 2 compared to 35 (9.5%) of group 1 (*p* = 0.007). **Conclusions:** More than 50% of older adults aged ≥ 75 years, diagnosed with CRC had evidence of anemia, at least 1 year before cancer diagnosis. Health policymakers should educate physicians about the importance of recognizing abnormal laboratory results, such as anemia, particularly within this often-overlooked group of older patients to increase early detection of CRC.

## 1. Introduction

Iron deficiency anemia (IDA) is a common clinical condition affecting both developing and developed countries at all ages [1,2]. According to the World Health Organization (WHO) anemia is defined as having hemoglobin (Hb) levels < 12 g/dL in women and <13 g/dL in men [3]. When a ferritin level below 45 mcg/dL is identified as well, the diagnosis of IDA can be determined [2]. IDA arises from three principal etiologies: inadequate dietary intake, reduced intestinal absorption, and chronic blood loss. Dietary insufficiency is common in individuals who adhere to vegan or vegetarian diets without supplementation. Malabsorption may occur in celiac disease, other intestinal disorders, or after gastrointestinal surgery. Chronic blood loss may stem from gastrointestinal bleeding due to malignancy, vascular lesions, or peptic ulcer disease, or from non-gastrointestinal causes such as repeated blood donation or menstrual bleeding in premenopausal women [2,4]. However, in adult men and postmenopausal women, IDA is usually caused by chronic occult gastrointestinal bleeding [2]. Therefore, once a diagnosis of IDA has been made, an investigation to localize the underlying pathological lesion should be done, usually by endoscopic evaluation [2]. Recently, a systematic review and meta-analysis of 39 studies (9807 patients with IDA) confirmed an overall prevalence of 8% for CRC and a prevalence of 5% for upper GI malignancy among IDA patients undergoing endoscopy [5]. Demb et al. showed that out of a large cohort of 239,000 US Veterans aged 18–49, IDA conferred a hazard ratio of 10.81 (95% CI, 8.25–14.33) for early-onset CRC [6]. However, recently, with the increase in life expectancy in Western populations, it has been recognized that aging is associated with increased incidence and prevalence of anemia [7]. On one hand, the older the population, the more frequently anemia is encountered [8,9]. On the other hand, according to the recent recommendation of U.S. Multi-Society Task Force (MSTF) [10,11], screening for CRC among adults aged 76–85 years is not universally recommended and adults above age 85 should not undergo screening at all [10,11]. Therefore, we investigated how frequently IDA and other clinical features appear before a CRC diagnosis in patients aged 75 years and above, compared to those aged 50–75 years with CRC, and assess which early signs support CRC screening in these older adults and should be the focus of general physicians.

## 2. Materials and Methods

### 2.1. Setting

We used a retrospective cohort of newly diagnosed cases of primary CRC that were surgically treated at our center (Rabin Medical Center, Beilinson Hospital), a tertiary care health system in Dan-Petach Tikva province belonging to Clalit Health Services (CHS), the largest Israeli Health Maintenance Organization (HMO). CHS provides comprehensive care (primary and tertiary) to nearly 5 million members nationwide [12]. The study was approved by the local Institutional Review Board.

### 2.2. Cohort Establishment

A list of consecutive patients that underwent surgery for CRC from 1 January 2014 to 31 December 2019 was obtained through the administrative coding data of the tertiary center. We included only patients who belonged to the CHS at least 36 months in continuity before cancer diagnosis and remained so up until January 2023 (or death). The continuity CHS membership from 36 months before cancer diagnosis and up to 1 January 2023 (or death) was assured by the transaction of the data through the CHS central database. The cohort was divided into two groups: group 1: adults aged from 50 to 75 years and group 2: older adults aged ≥ 75 years.

### 2.3. Primary CRC Diagnosis

Specific data on the tumor’s stage, location and histology at the operation time and death date were retrieved from the Israel National Cancer Registry (INCR). We linked the cohort to the INCR by way of the personal identification number given to all Israeli citizens at birth or immigration. The INCR, a population-based registry in operation since 1960, meets internationally accepted requirements for the coding system (International Classification of Diseases for Oncology, Third Edition) and completeness of data. Reporting has been mandatory since 1982; coverage exceeds 95% and has been excellent since the inception of the registry in the 1960s [13]. The INCR data includes personal identification, the date of diagnosis, site affected, the International Classification of Disease (ICD-O-3) [14] code of the tumor and histologic description of the tumor. We included only colorectal cancers with a histologic report of adenocarcinoma (codes 8000/3, 8001/3, 8010/3, 8140/3 and 8480/3) excluding all other histologic diagnosis. In addition, we excluded any recurrent CRC, using this method.

### 2.4. Data Collection

At the CHS hospitals, patients’ charts have been completely electronic (paperless) since the year 2000. The complete charts are available as PDF files and diagnoses are coded according to the ICD-O-3 [12]. We performed comprehensive chart review of in-hospital records of the admitting file when the surgery was performed. Demographic data including age, gender and ethnicity were extracted from the electronic medical records (EMR) of the patients. In addition, the route to diagnosis of the CRC was extracted and categorized into one of six options: symptoms (rectal bleeding, change in bowel habits or abdominal pain), positive fecal occult blood test (FOBT) (without symptoms), IDA, emergent operation due to colon obstruction or perforation, abnormal imaging, and screening for CRC (including patients who underwent colonoscopy because of family history of CRC).

The first day of definitive treatment was defined as the day of surgery for the CRC or the first oncology visit in the cases of rectal cancers that received neo-adjuvant treatment. Data for the date of surgery and the first outpatient oncology visit were extracted from the specific administrative data of the CHS.

Data during the 36 months preceding the first day of definitive treatment were extracted from the CHS headquarters database. At the CHS, all lab results, including the lab results of patients hospitalized in hospitals that belong to the CHS, are transmitted and stored at a central system. The central logistic system, which collects data from all CHS-approved pharmacies, provides information on the number of tablets each patient has taken. Both laboratory and pharmacy data are available since 1998. Laboratory parameters before the CRC definitive treatment, including hemoglobin level, iron level and ferritin level, together with the data regarding iron treatment, were extracted and categorized according to five iron deficiency anemia related laboratory events categories (L.E.C.):

1. Anemia at least 1 year prior to CRC diagnosis, which has been defined as blood Hb < 14 gr/dL for males (since according to the laboratories of CHS the normal lower limit of Hb level was 14 gr/dL, we used this value as the cutoff for defining anemia in men) and <12 gr/dL for females.
2. Severe anemia at least 1 year prior to CRC diagnosis defined as Hb < 12 g/dL for males and <10 g/dL for females.
3. Low iron level at least 1 year prior to CRC diagnosis defined as any serum iron level below the normal values of the performing laboratory.
4. Low ferritin level has been defined as below 15 ng/mL [2].
5. Iron treatment at least 1 year prior to CRC diagnosis defined as any purchased number of one of the iron supplementation treatments according to the ATC classification system definitions [15].

The time from the first time of each event in the preceding 36 months to the first day of treatment was calculated.

### 2.5. Statistical Analysis

A comparison between the two groups by using SPSS version 29 (IBM, Chicago, IL, USA) was conducted. Data was presented as mean ± standard deviations (SD) for continuous variables and as a percentage (%) of the total group for categorical variables. Univariate cox regression analysis to examine factors related to laboratory events of iron deficiency anemia was performed. A multivariate binary logistic regression model was used on the variables found to be significant in the univariate analysis. *p*-values less than 0.05 were considered statistically significant.

## 3. Results

**Figure 1:**
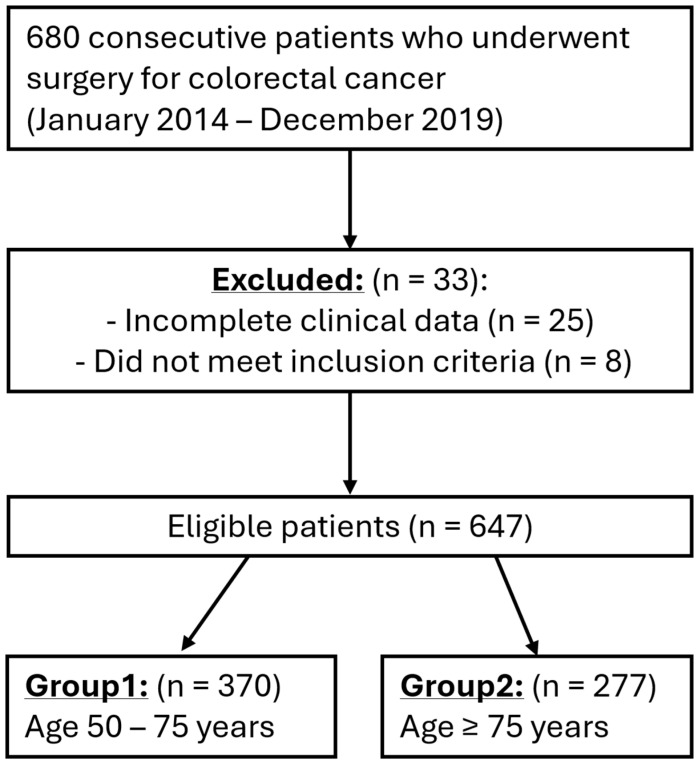
Flow-chart of the study cohort.

Between 1 January 2014, and 31 December 2019, 680 consecutive patients underwent surgery for colorectal cancer (CRC) diagnosis at our center. After excluding 33 patients due to incomplete clinical data or failure to meet the inclusion criteria, our cohort included 647 patients. All patients were members of the Clalit Health Services (CHS) for at least three years before their cancer diagnosis and remained enrolled up to December 2019 (unless deceased). The cohort was divided into two groups: Group 1—adults aged 50–75 years, Group 2—older adults aged ≥ 75. Group 1 included 370 patients (57.1%, mean age 65.8 ± 6.5 years), and Group 2 included 277 patients (42.8%, mean age 80.7 ± 4.1 years), as shown in Figure 1.

Table 1 shows the patient characteristics and routes to CRC diagnosis. Symptoms such as rectal bleeding, changes in bowel habits, and abdominal pain were the most common diagnostic routes in both groups. Investigation of IDA was more frequent among the patients of group 2 (20.6% vs. 11.6%, *p* = 0.002), while investigation following a positive FOBT was more frequent among the younger group.

Table 2 shows the characteristics of the colorectal cancers diagnosed in our cohort. No significant differences were found in the histology or the stage of cancer between the two groups. However, the tumors’ location differed significantly between the two groups. Right-sided (proximal) colon cancers were more prevalent among the older group (40.4% vs. 35.9%, *p* < 0.001), whereas rectal cancers were more frequent in the younger group (40.8% vs. 31.0%, *p* = 0.011).

Table 3 shows the incidence of iron deficiency anemia related laboratory events in Group 2 compared to Group 1. Anemia and severe anemia were higher among group 2 (56.3% vs. 38.4%, *p* < 0.001, and 20.2% vs. 10.3%, *p* < 0.001, respectively). Low serum iron and iron treatment were also higher among group 2 (17.7% vs. 9.5%, *p* = 0.002, and 23.1% vs. 16.5%, *p* = 0.035, respectively). Overall, 60.3% of the older and 43.8% of the younger group had any lab event (*p* < 0.001) (as shown in Table 3 and Table 4). A low ferritin was more common among the older group compared to the younger group (4.7% vs. 3.8%, *p* = 0.567), however this difference did not reach statistical significance. Overall, in both groups less than 5% of the patients had low ferritin more than 1-year prior to CRC diagnosis.

Univariate cox regression analysis to examine which factors were related to the laboratory events of iron deficiency anemia was performed. Results were significant for the male gender (*p* = 0.004), and for older age (*p* < 0.001), as shown in Table 4.

When we entered these factors into a multivariate binary logistic regression model, these factors remained significantly associated with the existence of L.E.C.: older age with an odds ratio (OR) of 1.96 (95% CI 1.43–2.70, *p* = 0.004), and a gender-adjusted OR of 1.59 (95% CI 1.16–2.18, *p* = 0.00). These results are shown in Table 5.

Laboratory events related to IDA were more prevalent among men compared to women (56.1% vs. 44.9%, *p* = 0.004), as shown in Table 4.

## 4. Discussion

The present study sought to investigate the frequency of anemia and other clinical features of CRC long before cancer diagnosis among older adults aged ≥ 75 years compared to patients aged 50–75 years. This study found that more than one fifth (20.6%) of older adults aged ≥ 75 years, who were diagnosed with CRC had IDA at least one year prior to their cancer diagnosis. In addition, when we investigated the frequency of any laboratory event related to iron deficiency, for example: iron treatment, low ferritin or iron level, etc., we demonstrated that laboratory events linked to IDA are more common in patients aged ≥ 75 years than in the younger group. Moreover, age ≥75 years doubles the risk for at least one IDA lab event compared to age below 75. These findings are consistent with the study by Shalata et al., which reported that among 724 older adults with CRC diagnosis, aged ≥ 70, IDA was the second most common cause (18.8%) leading to CRC diagnosis, following clinical symptoms such as abdominal pain, rectal bleeding, and changes in bowel habits [16]. When we investigated the frequency of low ferritin, we found that overall, in both groups less than 5% of the patients had low ferritin more than 1 year before the diagnosis. Since ferritin is an acute phase reactant which can increase during inflammation, infection, liver disease and obesity [17], we believe that its use as a sole red flag to CRC is less sensitive than using IDA itself. Additionally, when we investigated the prevalence of other symptoms or red flags that preceded CRC diagnosis, we found that in our study, similar to Shlata et al. (which demonstrated that 4.14% of the older patients with CRC are diagnosed following positive FOBT [16]), 4.7% of older adults, are diagnosed following FOBT compared to 15.4% in the lower age-group. Since, according to recent guidelines, a fecal immunohistochemical test is not recommended for CRC screening of average-risk individuals above 75 years [18] it is understandable that we demonstrated a higher prevalence of positive FOBT among the younger group.

Another important finding of our study is the significant difference in tumor location between the two groups. We demonstrated that right-sided (proximal) colon cancers were more prevalent among the older patients (40.4% vs. 35.9%, *p* < 0.001), whereas rectal cancers were more frequent in the younger group (40.8% vs. 31.0%, *p* = 0.011). These findings are consistent with the results of Abu Freha et al., who have also reported that right-sided colon cancers were more prevalent in the older group than in the younger group (43.9% vs. 33.7%, *p* < 0.001, respectively) [19]. This predominance of right colon cancers that we have found in the older group aligns with the existing literature that has suggested age-related differences in tumor location, between younger and older patients [11,20,21]. Specifically, younger patients are more likely to present with rectal cancer, while older patients tend to have right-sided colon cancer [22,23]. One explanation for this difference involves genetic changes associated with age, as discussed by Christenson et al. who showed that in CRC patients, aging was associated with higher rates of BRAF p. V600E mutations [24], a mutation that is usually common in right side colon cancers [25,26].

The tendency of right-sided tumors to bleed can help explain the association between anemia in older adults. Right-sided colon cancers are more likely to present with occult blood loss and iron deficiency anemia due to surface ulcerations of advanced tumors, which often bleed slowly and are less likely to cause visible hematochezia compared to left-sided tumors [27]. This occult bleeding can lead to significant blood loss over time, resulting in anemia, which is more commonly observed in older patients with right-sided colon cancers [28]. However, it is important to be aware that regardless of the possibility of blood loss from the gastrointestinal system, the prevalence of anemia is higher in older people, also due to chronic diseases [27,28,29,30,31].

The prevalence of IDA in elderly CRC patients, already present more than 1 year before the CRC diagnosis, as shown in our study, underscores the need for timely and thorough diagnostic evaluations. Studies have shown that early colonoscopy in patients with pre-existing IDA can improve overall survival by facilitating early diagnosis and treatment of CRC [20]. Nevertheless, sometimes, the presence of IDA in CRC patients is associated with higher rates of comorbidities that can complicate treatment and affect prognosis [19].

Our study also noted the differences in the stages of CRC between older and younger patients. However, although we found that in the younger group the rate of advanced stage of CRC was higher compared to the older group (34.3% vs. 28.2%), this difference did not reach statistical significance. Nonetheless, this tendency is consistent with previous studies that indicated that younger CRC patients often had later stage presentations [21,32].

In our analysis, we did not find differences in the rate of CRC among males and females in either group. However, male sex was a risk factor (1.5 times) for at least one IDA lab event, at least 1 years before CRC diagnosis. Gender difference in healthcare utilization, as shown previously [33,34], may explain this finding.

Recently, Demb et al., reported in a systematic review and meta-analysis which included 24,908,126 patients with early-onset CRC (diagnosis before the age of 50 years), that the most common presenting symptoms were: hematochezia, abdominal pain and changes in bowel habits [35]. In addition, the MSTF recently updated its recommendation on CRC screening of average-risk population and suggested expanding the age range to 45–75 without changing the upper age limit for CRC screening [11]. As a result, individuals aged 45–49, who were previously not included in routine screening recommendations are now recognized as an important group for early detection efforts. The uniqueness of our study lies in its focus on the older population, a group often underrepresented and under-reported in clinical research. By examining the prevalence of IDA in older adults with CRC, this study provides valuable insights that can inform clinical practice and improve patients’ outcomes [36].

These findings are consistent with national registry data showing that nearly one-third of male and almost 40% of female CRC cases occur in patients aged 75 years and older in Israel [37]. This emphasizes the need to refine screening policies and awareness in the older populations. Compared to other studies, such as those by Wilson et al. and Lachlan et al., which also highlighted the prevalence and implications of IDA in CRC patients, our study particularly addressed the older population, thereby filling a critical gap in the literature [38,39]. A further strength of our study is its comprehensive data coverage, which allowed us to identify any lab events related to anemia up to 36 months before CRC diagnosis. Older adults often encounter greater barriers to healthcare access, so our study emphasized the need of general physicians to be more aware of these challenges, and to follow up this specific population with further tests as timely identification of health issues can affect their overall prognosis and treatment outcomes [40,41].

Our study has several limitations. Its retrospective design and data extracted from a single tertiary hospital, may introduce selection bias, and the reliance on health maintenance organization (HMO) data may limit the generalizability of the findings. Additionally, the study does not account for potential confounding factors such as dietary habits [42,43], socioeconomic status [44,45], and access to healthcare [46,47], which could influence the prevalence of IDA and CRC. In addition, data regarding smoking status, a risk factor for CRC, was missing too [48]. Another limitation is the fact that we did not retrieve data regarding chronic comorbidities of the patients in both groups. However, since it is well known that anemia of chronic disease can mask iron deficiency anemia [49], we believe that our results, which demonstrated that anemia and its related red flags indicated CRC diagnosis even without adjustments to the chronic morbidities, strengthen the argument that anemia is an important red flag for CRC diagnosis and remains a strong predictor of CRC regardless of comorbidities, as it has been already demonstrated in previous studies [50,51].

Despite these limitations, the study’s large sample size and the use of robust statistical methods enhance its validity and reliability.

## 5. Conclusions

This study highlights an important gap in geriatric care, reflected in the clinical neglect of anemia related findings in older adults. Our data demonstrates that many older patients experienced IDA and other anemia related laboratory abnormalities for as long as two years before their CRC was eventually diagnosed, yet these abnormalities often did not prompt a timely diagnostic evaluation. This prolonged period of uninvestigated anemia underscores a missed opportunity for earlier detection in a population that already faces reduced access to screening and a higher prevalence of right-sided tumors. Enhancing clinical vigilance and adopting a more proactive approach to abnormal laboratory results in older adults may contribute to earlier CRC identification and improved outcomes.

## Figures and Tables

**Table 1:** Patient’s characteristics and route to diagnosis.

		Age Group (y ^a^)		Total	*p*
		50–75	≥75		
**Total**		370	277	647	
		100%	100%	100%	
**Age, yrs**	mean (sd ^b^)	65.8 (6.58)	80.7 (4.18)		<0.001
**Sex**	Men	198	148	346	0.983
		53.5%	53.4%	53.5%	
	Women	172	129	301	
		46.5%	46.6%	46.5%	
**Ethnicity**	Jew	353	275	628	0.0040
		95.4%	99.3%	97.1%	
	Arab	17	2	19	
		4.6%	0.7%	2.9%	
**Diagnosis**	Symptomatic ^c^	216	177	393	0.1558
		58.4%	63.9%	60.7%	
	Positive FOBT, asymptomatic	57	13	70	0.0000
		15.4%	4.7%	10.8%	
	Iron deficiency anemia	43	57	100	0.0020
		11.6%	20.6%	15.5%	
	Emergency operation	27	18	45	0.7004
		7.3%	6.5%	7.0%	
	Imaging abnormality	18	11	29	0.0004
		4.9%	4.0%	4.5%	
	FH ^d^-CRC ^e^	6	1	7	0.1430
		1.6%	0.4%	1.1%	
	Screening	3	0	3	0.3727
		0.8%	0.0%	0.5%	

^a^ yrs = years; ^b^ sd = standard deviation; symptomatic ^c^ = rectal bleeding, change in bowel habits, abdominal pain; ^d^ FH = family history; ^e^ CRC = colorectal cancer.

**Table 2:** Clinical and pathological characteristics of CRC ^a^ in the two age groups.

Age Groups (Years)		50–75	≥75	Total	*p*-Value
Total		370	277	647	
Location	Right	133 (35.9%)	112 (40.4%)	245 (37.9%)	<0.001
	Left	86 (23.2%)	79 (28.5%)	165 (25.5%)	0.13
	Rectum	151 (40.8%)	86 (31%)	237 (36.6%)	0.011
Histology	Non mucinous	323 (87.3%)	243 (87.7%)	566 (87.5%)	0.874
	Mucinous	47 (12.7%)	34 (12.3%)	81 (12.5%)	
Stage ^b^	I–II	243 (65.7%)	199 (71.8%)	442 (68.3%)	0.095
	III–IV	127 (34.3%)	78 (28.2%)	205 (31.7%)	

^a^ = CRC, colorectal cancer. ^b^ = stage was defined by the TNM system.

**Table 3:** Laboratory characteristics of the study cohort in the year preceding diagnosis.

Age Groups (Years)		50–75	≥75	Total	*p*-Value
***n***		370	277	647	
**Anemia > 1 y ^a^**	No	228 (61.6%)	121 (43.7%)	349 (53.9%)	<0.001
	Yes	142 (38.4%)	156 (56.3%)	298 (46.1%)	
**Severe anemia > 1 y ^a^**	No	332 (89.7%)	221 (79.8%)	553 (85.5%)	<0.001
	Yes	38 (10.3%)	56 (20.2%)	94 (14.5%)	
**Low iron > 1 y ^a^**	No	335 (90.5%)	228 (82.3%)	563 (87%)	0.002
	Yes	35 (9.5%)	49 (17.6%)	84 (13%)	
**Low ferritin > 1 y ^a^**	No	356 (96.2%)	264 (95.3%)	620 (95.8%)	0.567
	Yes	14 (3.8%)	13 (4.7%)	27 (4.2%)	
**Iron supplemental > 1 y ^a^**	No	309 (83.5%)	213 (76.9%)	522 (80.7%)	0.035
	Yes	61 (16.5%)	64 (23.1%)	125 (19.3%)	
**The number of laboratory events that exist**	0	208 (56.2%)	110 (39.7%)	318 (49.1%)	<0.001
	1	97 (26.2%)	78 (28.2%)	175 (27%)	
	2	30 (8.1%)	40 (14.4%)	70 (10.8%)	
	3	14 (3.8%)	26 (9.4%)	40 (6.2%)	
	4	14 (3.8%)	13 (4.7%)	27 (4.2%)	
	5	7 (1.9%)	10 (3.6%)	17 (2.6%)	
**Existence of any laboratory event category ^b^**	No	208 (56.2%)	110 (39.7%)	318 (49.1%)	<0.001
	Yes	162 (43.8%)	167 (60.3%)	329 (50.9%)	

^a^ = y, year; ^b^ = laboratory events include anemia, severe anemia, low iron, low ferritin, and iron supplementation.

**Table 4:** Univariate analysis of factors associated with existence L.E.C. ^a^.

		Total	Gender		*p*-Value	Age		*p*-Value	Family History		*p*-Value
			Men	Women		50–75 y ^b^	≥75 y ^b^		No	Yes	
**L.E.C. ^a^**	No	318 (49.1%)	152 (43.9%)	166 (55.1%)	0.004	208 (56.2%)	110 (39.7%)	<0.001	324 (50.62%)	2 (28.57%)	0.740
	Yes	329 (50.9%)	194 (56.1%)	135 (44.9%)		162 (43.8%)	167 (60.3%)		316 (49.37%)	5 (71.42%)	
**Total**		647 (100%)	346 (100%)	301 (100%)		370 (100%)	277 (100%)		640 (100%)	7 (100%)	

^a^ = L.E.C., Laboratory Event Category; ^b^ = y, years.

**Table 5:** Factors associated with existence of L.E.C. ^a^ on multivariate binary logistic regression model.

	Odds Ratio	95% Confidence Interval	*p*-Value
Male gender	1.59	1.16–2.18	0.004
Age (years)	1.967	1.431–2.706	<0.001

^a^ = L.E.C., Laboratory Event Category.

## Data Availability

The raw data supporting the conclusions of this article will be made available by the corresponding author on request.

## Abbreviations

The following abbreviations are used in this manuscript:

CRC	Colorectal cancer
HMO	Health maintenance organization
IDA	Iron deficiency anemia
WHO	World Health Organization
MSTF	Multi Society Task Force
CHS	Clalit health organization
INCR	Israel National Cancer Registry
ICD-O	International Classification of Disease
EMR	Electronic medical records
FOBT	Fecal occult blood test
L.E.C	Laboratory event category
SD	Standard deviation
OR	Odds ratio
